# Orthognathic surgery and aligners. A comparative assessment of periodontal health and quality of life in postsurgical orthodontic treatment with aligners versus traditional fixed appliances: a randomized controlled trial

**DOI:** 10.4317/medoral.25555

**Published:** 2023-04-07

**Authors:** Patricia de Leyva, Jose Miguel Eslava, Federico Hernández-Alfaro, Julio Acero

**Affiliations:** 1 Department of Oral and Maxillofacial Surgery, Ramón y Cajal University Hospital, Madrid, Spain.; 2 Department of Oral and Maxillofacial Surgery, Centro Médico Teknon, Barcelona, Spain

## Abstract

**Background:**

Removable clear aligners have become very popular in the last few decades, but they are still little used in the field of orthognathic surgery (OS). The objective of this study was to compare periodontal health and quality of life (QoL) associated to postsurgical orthodontic treatment.

**Material and Methods:**

Patients with dentofacial deformities undergoing OS were randomly allocated to receive postsurgical orthodontic treatment with either fixed orthodontic appliances or Invisalign. The main outcomes were periodontal health and QoL. Plaque index, probing depth and bleeding on probing were assessed as periodontal health indicators. QoL was assessed through the Orthognathic Quality of Life Questionnaire (OQLQ-22) and the Oral Health Impact Profile (OHIP-14). Data were analyzed before surgery and end of treatment. Total duration of treatment was also recorded.

**Results:**

Twenty-eight patients were randomized, (16 women, 12 men). Periodontal assessment showed better outcomes for the Invisalign group: bleeding on probing (*p*=0.013), plaque index (*p*=0.001) and probing depth (*p*<0.001). The QoL questionnaires showed significant differences in favor of the Invisalign group: OHIP-14 (*p*=0.004) and OQLQ-22 (*p*=0.002). Total duration of treatment was similar in both groups (*p*=0.575).

**Conclusions:**

Compared to traditional orthodontics with fixed appliances, patients managed with clear aligners after OS (surgery-first approach) had better periodontal health and QoL outcomes.

** Key words:**Invisalign, surgery first, orthognathic surgery, periodontal health, quality of life.

## Introduction

Dentofacial deformities are a consequence of discrepancies in the shape and size of the jaws, leading to dental malocclusions and facial disharmony. Orthognathic surgery (OS), through a combined orthodontic-surgical approach, is able to manage such skeletal discrepancies, improving facial aesthetics, occlusion and airway volume. The traditional protocols for treating dentofacial deformities are based on the use of pre- and postsurgical orthodontic treatment. In contrast, surgery-first protocols produce immediate improvement of the facial profile and airway volume, and a noticeable reduction in total treatment duration (1,2). The reliability of this technique in terms of long-term stability and the shortening of total treatment time has been confirmed in previous studies (2,3).

On the other hand, fixed orthodontic devices such as brackets complicate oral hygiene, potentially leading to plaque accumulation and periodontal disease (4). Besides, many adult patients feel unconfident or even ashamed of wearing brackets in public (5). This is the main reason why clear aligners such as Invisalign (Align Technology, Santa Clara, CA) have become so popular in the last few decades for nonsurgical orthodontic treatments (6).

Many articles have evaluated changes in quality of life (QoL) after OS and its functional and psychosocial benefits for patients with dentofacial deformities (7-9). Patient satisfaction and periodontal health status during isolated orthodontic treatment with fixed brackets versus removable aligners have also been evaluated (10-13). A good indicator of the existing concern on this matter is the fact that some new papers have been published lately on OS and removable aligners (14-18). Kankam *et al*. stated the high demand for OS procedures, and their concern on the poor tolerance to conventional fixed appliances in some patients. As Chang *et al*. reported in their paper, many patients seeking OS treatment are adults who wish to avoid deterioration in their profile and facial esthetics during presurgical orthodontics. Thus, the combination of OS with clear aligners in a surgery-first protocol seems to be a win-win option.

The aim of the present study was to comparatively assess periodontal health and QoL among patients with dentofacial deformities combining the surgery-first approach with Invisalign versus fixed appliances. Our hypothesis was that patients in the Invisalign group would have a better periodontal health and higher satisfaction than those in the brackets group.

## Material and Methods

- Trial design

We report a 2-arm parallel, randomized, clinical trial with an allocation ratio of 1:1. The study was approved by the Institutional Review Board at our hospital (IRB number 095/17). This study followed the Declaration of Helsinki on medical protocols and ethics. No changes to the methods after trial commencement occurred. The presentation of this report is according to the CONSORT guidelines for reporting trials.

- Participants

The participants were recruited from the outpatient clinic at the craniofacial department of Ramón y Cajal University Hospital, University of Alcalá de Henares, Madrid, Spain. The surgeons in the craniofacial team were responsible for recruitment. Eligibility criteria were: 1) skeletal malocclusion requiring combined surgical and orthodontic treatment without extractions; and 2) informed consent for this novel protocol. The exclusion criteria were: 1) temporomandibular joint disorders or severe symptoms; 2) uncontrolled periodontal disease; 3) severe crowding requiring extractions; 4) class II division 2 malocclusion with overbite or severely altered curves of Spee; or 5) severe asymmetry.

- Interventions

Baseline characteristics including periodontal status were recorded. After recorded, all patients in the study received professional oral hygiene treatment by an experienced dental hygienist. They were also instructed on the same standardized oral hygiene protocol before and during orthodontic treatment. This included the proper use of toothbrush and interdental brushes three times daily. Baseline data recording and hygiene treatment took place before random allocation.

Participants were randomly assigned to postsurgical orthodontic treatment with either clear aligners (Invisalign group) or fixed appliances (brackets group). All patients were eligible for a surgery-first OS approach. The Invisalign system was used in all clear aligner cases. All surgical procedures were performed by the same surgical team and all patients were treated by an Invisalign certified orthodontist with experience in OS. All patients signed an informed consent document after questions and concerns were clarified by the surgical team.

All patients had an appointment with the dental hygienist for professional tooth cleaning after recruitment. The initial diagnostic work-up, preoperative planning, orthodontic preparation and surgical execution proceeded according to our standardized protocol for surgery-first orthognathic procedures. Patients allocated to the brackets group underwent a preoperative orthodontic appointment one week prior to surgery for bracket bonding. Patients in the Invisalign group undergoing non-segmented maxillary surgery had an orthodontic appointment one week before surgery for intraoral scanning in order to fabricate the aligners.

The patients were operated upon under general anesthesia. Single jaw or bimaxillary surgery was performed, depending on the individualized treatment plan. For patients in the Invisalign group, 4 to 8 transmucosal 2.0 mm screws were placed before incision. If the maxilla needed segmentation, 8 screws were placed; in one-piece maxillas, 4 screws were used. Surgical protocol was the same for both groups. The duration of surgery was recorded.

All patients had the first appointment with the orthodontist one week after surgery. The brackets group began orthodontic treatment following that appointment. Patients in the Invisalign group and with segmented maxillary surgery were scanned within the first week and started using the aligners in the second week after surgery. Patients in the Invisalign group with non-segmented maxillary surgery began to use the first aligner within the first 10 days after surgery. The finishing criteria were the same for each group. They were established following our standard practice, in accordance with the American Board of Orthodontics Objective Grading System.

- Outcomes

Patient periodontal health status was evaluated based on the plaque index, probing depth and bleeding on probing. The plaque index was assessed from 0 to 3 by grading plaque accumulation in the gingival area according to the Ramfjord scale. The highest score was assigned to each sextant and the median value was selected for each patient. Probing depth was recorded with the periodontal probe. The probing sites were the mesiovestibular surface of the upper left central incisor and the lower right first molar. Bleeding on probing was registered 20 seconds after probing, and recorded as either absent or present. These parameters were recorded at three time points: at initial assessment after professional dental cleaning (baseline), 1 month after surgery (T1) and at follow-up evaluation at the end of orthodontic treatment, following removal of the brackets or last aligner (T2). This periodontal assessment protocol was designed following those reported in previous studies (4,13).

Two QoL questionnaires were used to assess patient satisfaction: the 14‑item short version of the Oral Health Impact Profile (OHIP‑14) and the 22‑item Orthognathic Quality of Life Questionnaire (OQLQ‑22). Pain intensity was assessed based on a 10‑point numerical rating scale (NRS). These parameters were recorded after bracket bonding for the brackets group patients and immediately before surgery (T0) and at T1 and T2.

The primary outcome measures were dental plaque as assessed with the plaque index and QoL related to OS as assessed with the Orthognathic Quality of Life Questionnaire at the 3 time points. The secondary outcomes were probing depth, bleeding on probing and QoL related to general oral health as assessed with the Oral Health Impact Profile questionnaire.

- Sample size

Sample size calculation was set to detect differences of 30% between groups for the main outcome by Mann-Whitney test. Standard deviation of 0.5 and 0.8 were assumed based on a pilot study of 10 patients. A minimum of 28 patients (14 per group) was needed in order to achieve 80% power at the 5% significance level.

- Randomization (sequence generation, allocation concealment and implementation)

Randomization was accomplished by the principal investigator (P.L.) by using an online software program (http://www.graphpad.com/quickcalcs/index.cfm), independent of the clinical investigator involved in recruitment (JM.E.) The program was set to provide a 1:1 allocation ratio, and automatically generated a randomization plan for treatment assignment.

**Figure 1:**
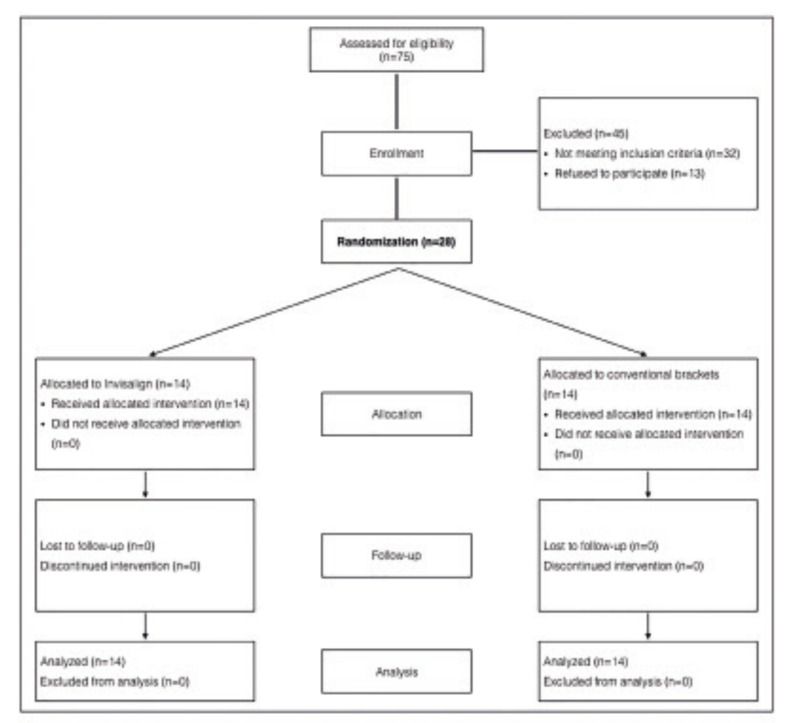
Flowchart showing recruitment of patients and allocation.

Allocation concealment was achieved with sequentially numbered, opaque, sealed envelopes, containing the treatment allocation cards. Envelopes were prepared before commencement of the trial, and they were kept locked and safe. The orthodontist was responsible for implementing the randomization process. This sequence is shown in Fig. 1.

- Statistical analysis

The analyses comprised observed data; there was no missing data. Continuous and categorical variables were reported as means and quartiles, and as numbers and frequencies, respectively. Cross-sectional between-group comparisons were made using the Student t-test or Mann-Whitney U-test (for continuous data with or without a normal distribution), or the Pearson chi‑squared test or Fisher exact test (for categorical data). A significance level of 5% (alpha = 0.05) was used. The SPSS version 19 statistical package was used for the study.

## Results

- Participant flow and recruitment

Twenty-eight patients (median age 28 years; range, 18-52 years) were randomized in a 1:1 ratio to either Invisalign or brackets. Patient recruitment commenced in May 2016 and ended in February 2017. No patients were lost to follow-up.

- Baseline data

Sixteen women and 12 men participated in the study. The Invisalign group comprised 14 patients (9 women and 5 men) with a median age of 26.5 years (interquartile range, 19; range 19-52). The brackets group comprised 14 patients (7 women and 7 men) with a median age of 28.5 years (interquartile range, 17; range 18-47). Baseline characteristics of the patients in the treatment groups are displayed in Table 1. Table 2 shows the demographic data of the patients, the protocol, diagnosis and type of surgery they received, and the duration of surgery and orthodontic treatment. These baseline data were not significantly different between the two groups.

**Table 1:**
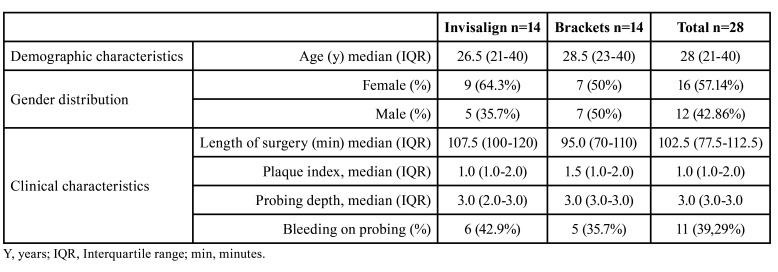
Baseline demographic and clinical characteristics of the randomized groups.

**Table 2:**
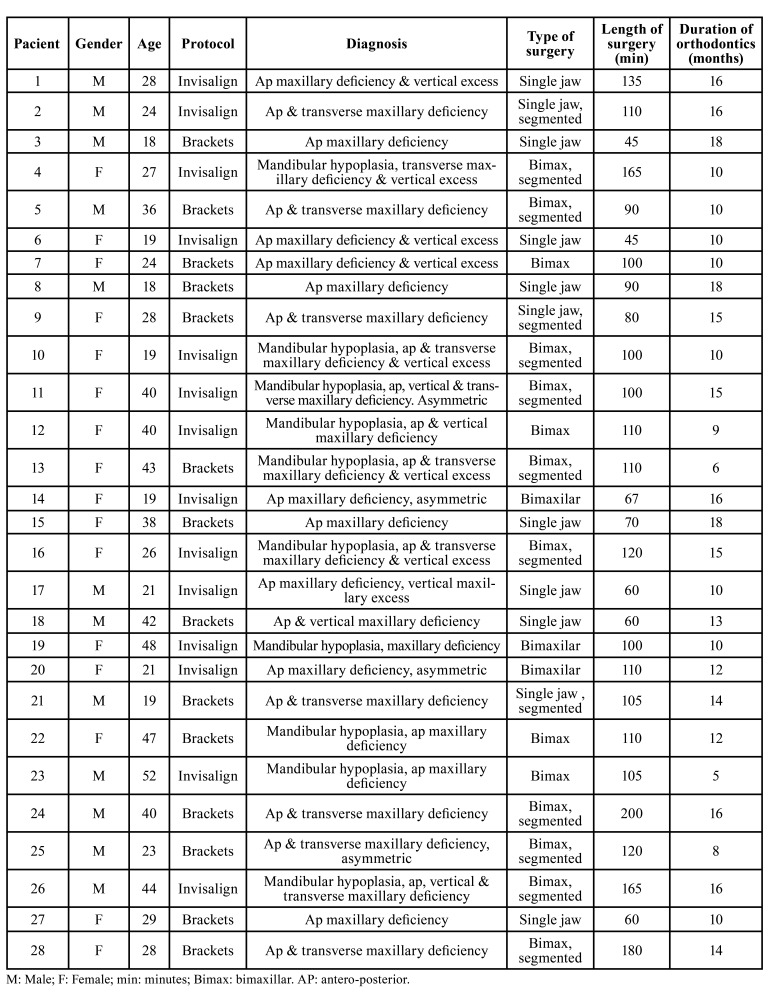
Demographic data of the patients, protocol, diagnosis and type of surgery, duration of surgery and orthodontic treatment.

Eight patients underwent single-jaw surgery; 3 patients had single-jaw, segmented surgery; 7 patients had bimaxillary surgery and 10 patients had bimaxillary, segmented surgery.

All randomized subjects were included in the analysis. No patients were lost to follow-up in any of the two groups.

- Outcomes

Periodontal outcomes:

Mean and standard deviation (SD) and median and quartiles of plaque index at T1 and T2 for each group, and their *p* value are shown in Table 3. Plaque index was lower in the Invisalign group at the two time points. This difference was statistically significant at T2 (*p*=0.001).

Mean and SD and median and quartiles of probing depth at T1 and T2 for each group, and their *p* value are shown in Table 3 as well. Probing depth was significantly lower in the Invisalign group at the two time points. Differences in favor of the Invisalign group were greater at T2 (*p*<0.001).

Bleeding on probing is shown as frequencies and percentages at the evaluation time points (Supplement 1). These frequencies were very similar between groups of treatment at T1 (*p*=0.699), but statistically different at T2, when only 1 patient presented positive bleeding in the Invisalign group, in comparison with 8 patients in the brackets group (*p*=0.013).

Quality of Life outcomes (Table 4):

The OHIP-14 results evidence a difference between the two groups, present already at T0, and that persisted throughout the study, reaching statistical significance at T2, demonstrating better QoL in the Invisalign group.

**Table 3:**
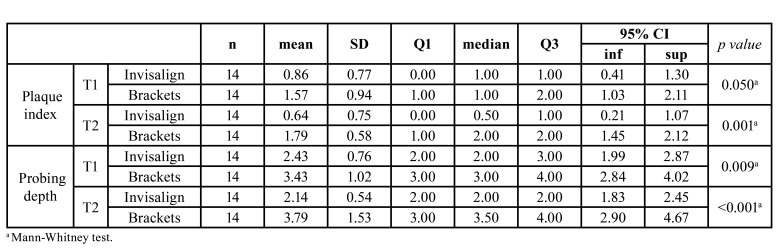
Plaque index and probing depth.

**Table 4:**
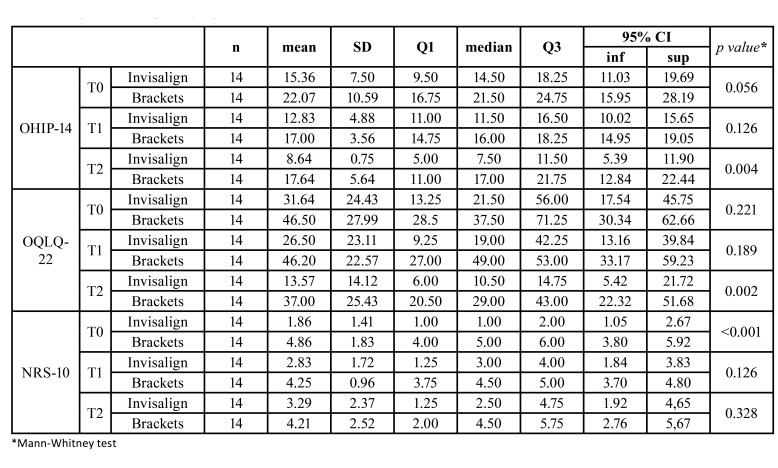
OHIP-14, OQLQ-22 and NRS-10 questionnaires.

**Figure 2:**
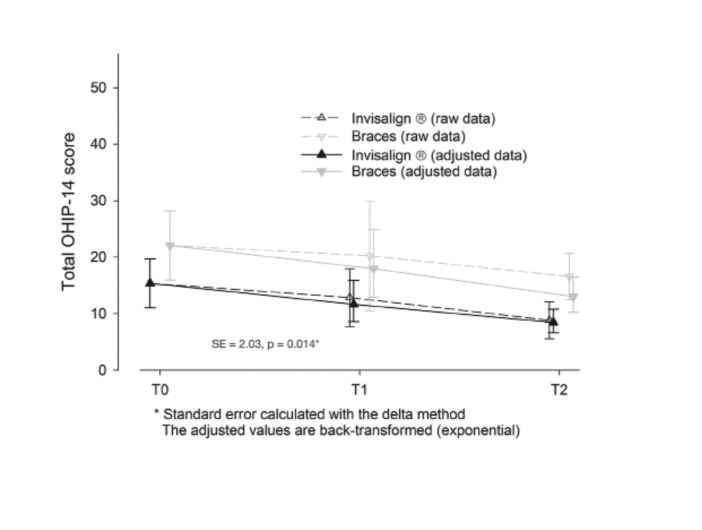
OHIP-14 Questionnaire.

The scores of the Invisalign group are below those of the brackets group (meaning a better QoL in the Invisalign group), a significant decrease is seen along the appointments for all the sample (OS treatment improves QoL/patients’ satisfaction) as well as a significant association with basal scores (the higher the basal score, the better scores in following appointments). The confidence intervals of the adjusted means clearly overlap at T1; at T2 overlapping is minimal: the difference is almost significant. However, the interaction treatment*appointment is not significant, therefore it is not possible to state that the difference between groups changes significantly between T1 and T2. This is interpreted as there is a significant difference, already present at baseline and maintained along the study. Moreover, the scores significantly decrease along the study (Fig. 2).

The OQLQ-22 results were better in the Invisalign group at all evaluations. The difference in favor of the Invisalign group proved statistically significant at T2 (meaning a better QoL in this group). The scores of the Invisalign group are below those of the brackets group, and a significant decrease along the appointments for all the sample (improve in QoL with treatment and during its course) and a significant association with basal scores (the higher the basal score, the better scores in following appointments) can be noticed.

**Figure 3:**
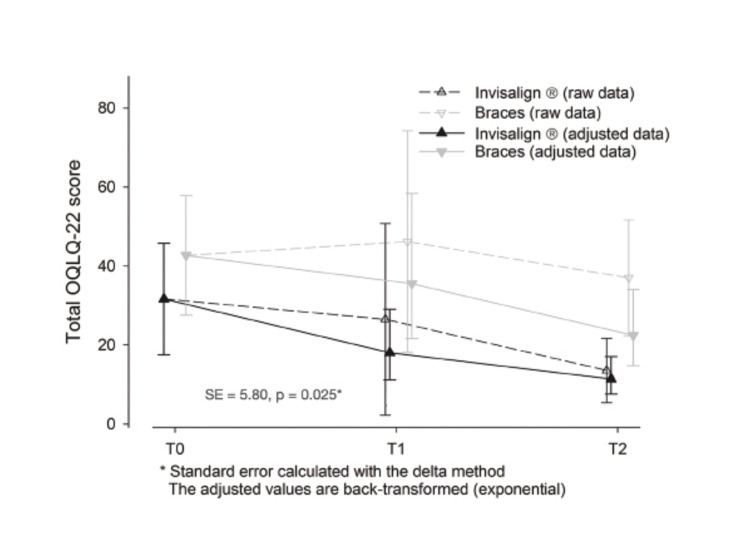
OQLQ-22 Questionnaire.

Although there is a net impact of treatment, confidence intervals overlap for all appointments. The interaction treatment*visit is not significant and the difference between groups does not change significantly between T1 and T2. This is interpreted as there is a significant difference, already present at baseline and maintained along the study. Moreover, the scores significantly decrease along the study (Fig. 3).

**Figure 4:**
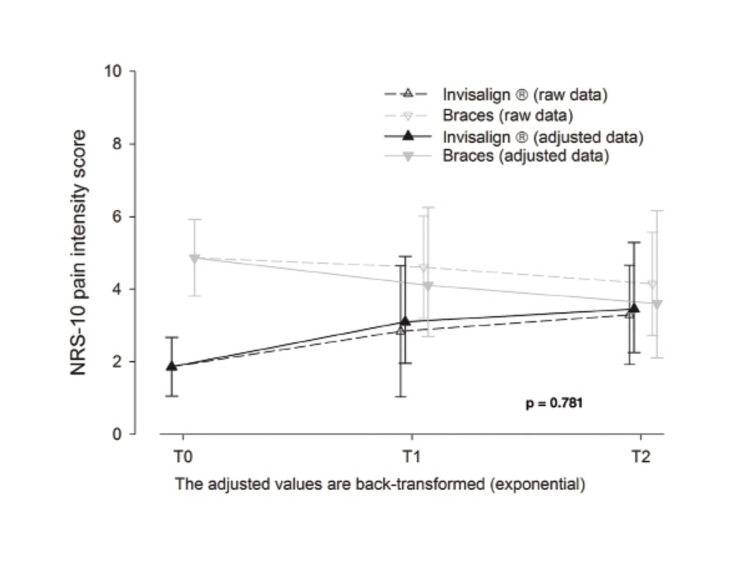
Pain intensity (NRS-10).

The pain intensity scores were lower in the Invisalign group at all evaluations. These differences proved statistically significant at T0 (Fig. 4).

The duration of treatment was very similar in both groups, with a mean duration of 12.14 months in the Invisalign group (range 5-16) and 13 months in the brackets group (range 6-18) (*p*=0.575).

## Discussion

- Limitations and generalizability

This study’s major limitation is the relatively small sample size, potentially reducing the statistical power. However, statistically significant differences were proven for the main outcome. Since candidates where not excluded regarding their age, or their type or severity of dentofacial malocclusion, this study closely simulates the real daily clinical practice. This is a strength of this study and allows for applicability of our treatment protocol to other settings and populations.

- Interpretation

Orthognathic surgery is the mainstay of treatment for dentofacial deformities. The aim of our study was to determine whether OS in combination with clear aligners affords advantages in terms of periodontal health and patient satisfaction. Our study found advantages of a clear aligner-based orthodontic treatment compared with fixed appliances in terms of QoL, pain intensity and periodontal health among patients with dentofacial deformities treated with a surgery-first protocol.

Oral health:

The beneficial effects of clear aligners lie in their intrinsic characteristic of being removable, and therefore in the easiness of tooth cleaning. Fixed orthodontic appliances make tooth cleaning slower and more difficult, usually leading to greater plaque accumulation. Different studies have compared fixed appliances versus clear aligners in isolated orthodontic treatment (4,10,13). Our study recorded overall better periodontal outcomes in the Invisalign group. Bleeding on probing was significantly different between the two groups at T2. The plaque index was also significantly different at T2, with increasing values in the brackets group over the course of treatment and sTable values in the Invisalign group. The latter group also obtained better results in terms of bleeding on probing, and the differences between the groups were significant at all three time points and greater at T2. Globally, the periodontal parameters remained sTable in the Invisalign group and slowly but progressively worsened in the brackets group. These results are similar to those reported in other studies comparing clear aligners and fixed appliances in orthodontics-alone treatments. The greater difficulty of tooth brushing and the impossibility of using dental floss, together with the swelling and soreness derived from surgery, result in progressive worsening of the periodontal status of those patients wearing brackets.

Patient satisfaction:

Different authors have concluded that, apart from objectively improving facial appearance and dental occlusion, orthognathic surgery can improve patient QoL. The changes in QoL during and after orthognathic surgery have been widely studied in the last few decades (9,19,20). In our study we compared a group of patients undergoing postsurgical orthodontic treatment with clear aligners and a group of patients receiving postsurgical orthodontic treatment with traditional fixed appliances. The surgical protocol was the same in both groups. Data from the questionnaires show better results in the Invisalign group from the beginning of the study (T0) and these differences reach statistical significance at T2 in both OHIP-14 and OQLQ-22 instruments. A lesser impact upon the daily life of the patients was therefore evidenced in the Invisalign group. We found benefits associated to Invisalign with both specific and generic QoL measures. The benefits in terms of QoL in our study were consistent with the findings of previous reports comparing QoL between Invisalign and fixed orthodontic appliances in isolated orthodontic treatment (12). Likewise supporting the QoL benefits in the Invisalign group is the fact that patients who received fixed appliances experienced more pain at T0, as evidenced by the results of the numerical rating scale, with statistically significant difference at this time point. Such differences are attributed to bracket bonding, which only concerned the patients in the brackets group. Besides, in our protocol, patients start orthodontic treatment 2-3 weeks after surgery. This means that the patients in the Invisalign group spend the first 2-3 weeks after surgery without the aligners, while patients in the other group wear the brackets and surgical wires during the worst weeks of the postoperative period, when swelling is at its peak. This may be an additional advantage of the Invisalign protocol.

In our study, the duration of treatment was very similar in both groups, and the difference was not statistically different. All patients in both groups finished their treatment uneventfully and with very satisfactory esthetic and functional outcomes. These results remained sTable at four-year follow-up.

Dentofacial deformities have an important psychosocial impact upon patients. The orthodontic phase of the ortho-surgical treatment may be regarded by patients as an important reason for avoiding correction of their problem. Although the present study is limited by the relatively small number of patients, our surgery-first and clear aligners protocol obtained significantly better outcomes in the periodontal assessment and in the quality of life questionnaires. Further RCT with a larger sample size are required to confirm these findings, but we believe that the possibility of offering clear aligner treatment to our orthognathic surgery patients, may play a major role in consolidating patient acceptance and cooperation during treatment, especially in adults.
